# MBD3 inhibits formation of liver cancer stem cells

**DOI:** 10.18632/oncotarget.13496

**Published:** 2016-11-22

**Authors:** Ruizhi Li, Qihua He, Shuo Han, Mingzhi Zhang, Jinwen Liu, Ming Su, Shiruo Wei, Xuan Wang, Li Shen

**Affiliations:** ^1^ Stem Cell Research Center, Department of Cell Biology, School of Basic Medical Sciences, Peking University, Haidian District, Beijing, 100191, China; ^2^ Beijing DongFang YaMei Gene Science and Technology Research Institute, Beijing, People's Republic of China; ^3^ State Key Laboratory of Organ Failure Research, Co-Innovation Center for Organ Failure Research, Guangdong Provincial Southern Medical University, Guangzhou, Guangdong Province, People's Republic of China

**Keywords:** induced liver cancer stem cells, MBD3/NuRD, c-JUN, C3A, transcriptional regulation

## Abstract

Liver cancer cells can be reprogrammed into induced cancer stem cells (iCSCs) by exogenous expression of the reprogramming transcription factors Oct4, Sox2, Klf4 and c-Myc (OSKM). The nucleosome remodeling and deacetylase (NuRD) complex is essential for reprogramming somatic cells. In this study, we investigated the function of NuRD in the induction of liver CSCs. We showed that suppression of methyl-CpG binding domain protein 3 (MBD3), a core subunit of the NuRD repressor complex, together with OSKM transduction, induces conversion of liver cancer cells into stem-like cells. Expression of the transcription factor c-JUN is increased in MBD3-depleted iCSCs, and c-JUN activates endogenous pluripotent genes and regulates iCSC-related genes. These results indicate that MBD3/NuRD inhibits the induction of iCSCs, while c-JUN facilitates the generation of CSC-like properties. The iCSC reprogramming approach devised here provides a novel platform for dissection of the disordered signaling in liver CSCs. In addition, our results indicate that c-JUN may serve as a potential target for liver cancer therapy.

## INTRODUCTION

Reprogramming of somatic cells by Yamanaka factors offers a new method to investigate cancer stem cells (CSCs). The reprogramming technique not only provides a way to induce CSCs from cancer cells, but also offers insight into the discovery of their disordered signaling pathways since many of the reprogramming factors are oncogenes. Therefore, reprogramming cancer cells to induced cancer stem cells (iCSCs) by Yamanaka factors profoundly affects cancer diagnosis and therapies.

Hepatocellular carcinoma (HCC) is the fifth leading cause of cancer-related death worldwide [1]. Liver cancer initiation and development resemble induction of induced pluripotent stem cells (iPSCs). HCC contains a subpopulation of stem-like cells expressing assorted stem cell markers [2]. Stem cell specific transcription factors, such as octamer-binding transcription factor 4 (Oct4) and Nanog are important regulators of CSCs maintenance and development, and play important roles in self-renewal, chemoresistance and invasion capacity [3, 4].

Methyl-CpG binding domain protein 3 (MBD3) is an essential subunit of the Nucleosome Remodeling and Deacetylase (NuRD) complex, without which the complex is not assembled [5]. A previous study has shown that MBD3 inhibits induction of iPSCs [6], and MBD3 depletion results in deterministic and synchronized iPSCs reprogramming with nearly 100% efficiency [7]. However, another study has reported that MBD3 facilitates reprogramming in a context-dependent manner [8]. More studies are needed to investigate the specific role of MBD3/NuRD in somatic cell reprogramming. In addition, understanding the MBD3 involvement in iCSCs generation is incomplete. Therefore, more studies are needed to understand the function of MBD3 in somatic and cancer cell reprogramming.

In this study, we investigated the role of MBD3/NuRD in liver iCSCs generation. Our results indicate that MBD3 inhibits the formation of liver CSCs. Furthermore, our results suggest that expression and activity of the transcription factor c-JUN are increased in iCSCs, and are essential for stemness and CSCs properties, indicating that c-JUN might serve as a target for liver cancer therapy.

## RESULTS

### Generation and characterization of iCSCs from C3A cells

**Figure 1:**
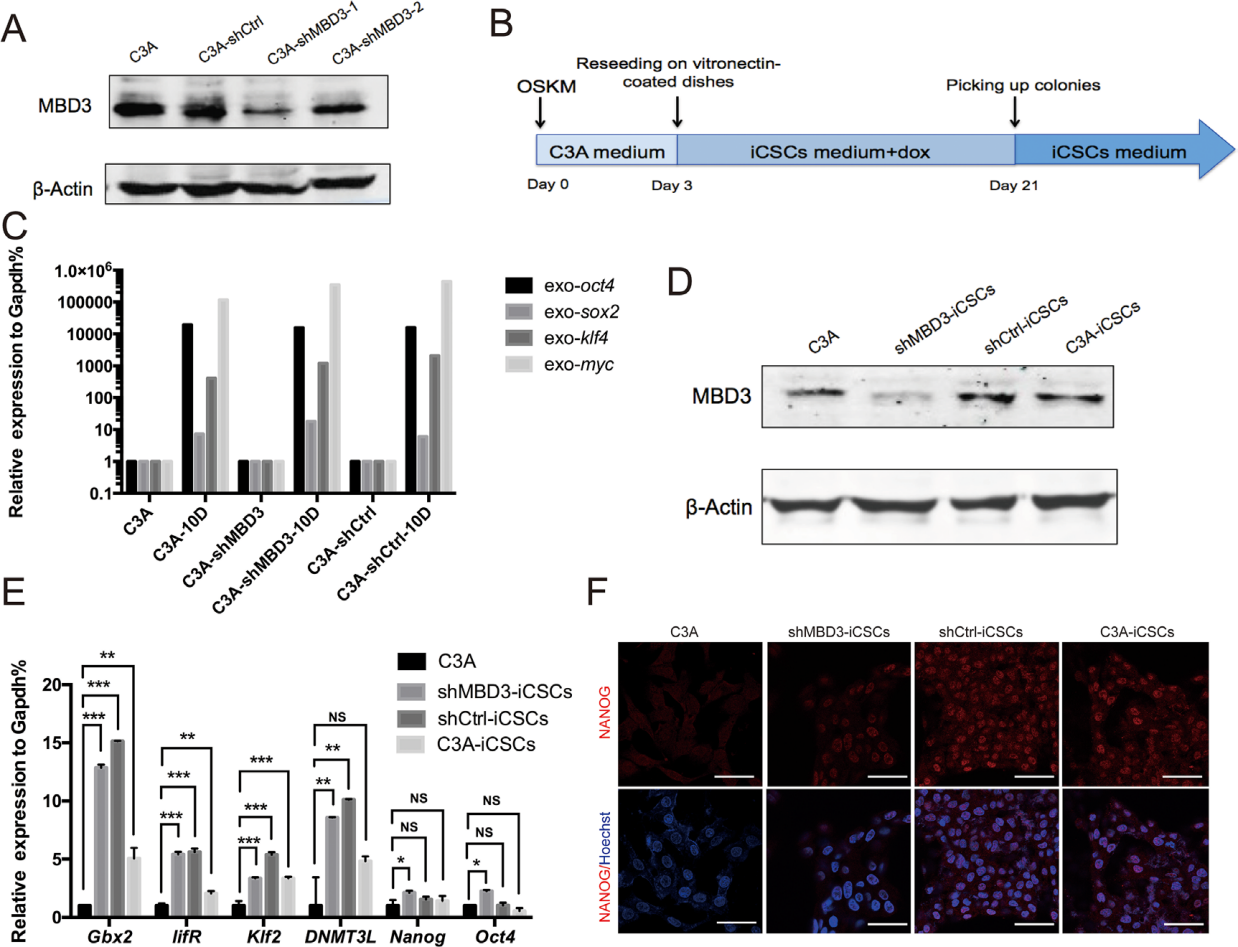
Generation of iCSCs by OSKM reprogramming **A**. Immunoblotting analysis of MBD3 in C3A cells, C3A-shCtrl cells, C3A-shMBD3-1 cells, and C3A-shMBD3-2 cells. **B**. Schedule of C3A-shMBD3, C3A-shCtrl, and C3A cells reprogramming into shMBD3-iCSCs, shCtrl-iCSCs, and C3A-iCSCs. **C**. RT-qPCR of exogenous *Oct4, Sox2, Klf4* and *c-Myc* in C3A cells, C3A-shMBD3 cells, and C3A-shCtrl cells on day 10 during reprogramming. **D**. Immunoblotting analysis of MBD3 in C3A cells, shMBD3-iCSCs, shCtrl-iCSCs and C3A-iCSCs. **E**. Relative expression of endogenous *Gbx2, lifR, Klf2, DNMT3L, Nanog* and *Oct4* in C3A cells, shMBD3-iCSCs, shCtrl-iCSCs and C3A-iCSCs analyzed by RT-qPCR. (n=3; *p<0.05, **p<0.01,***p<0.001, NS: no significant differences) **F**. Immunostaining of NANOG in C3A, shMBD3-iCSCs, shCtrl-iCSCs, and C3A-iCSCs. Scale bars, 50μm.

To investigate the role of MBD3 in iCSCs generation, we infected C3A cells with lentiviruses encoding shRNA-MBD3 or shRNA-Ctrl, and G418-resistant cells were obtained and identified. Figure 1A shows that MDB3 expression was suppressed in shRNA-MBD3-1 transfected cells. The stably transfected C3A-shMBD3 and C3A-shCtrl were used for reprogramming. We infected C3A-shMBD3, C3A-shCtrl and C3A cells with retroviruses encoding the transcription factors *Oct4, Sox2, Klf4* and *c-Myc* (OSKM) to induce reprogramming (Figure 1B). Ten days after infection, we confirmed the exogenous OSKM gene expression by qRT-PCR (Figure 1C). On day 21, single clones were picked and passaged on matrigel-coated dishes. The clones obtained from C3A-shMBD3, C3A-shCtrl, and C3A cells were named shMBD3-iCSCs, shCtrl-iCSCs, and C3A-iCSCs, respectively. MBD3 protein levels in iCSCs were analyzed by Western blotting. MBD3 expression was increased in shCtrl-iCSCs and C3A-iCSCs, while it remained negligible in shMBD3-iCSCs (Figure 1D). Next, we measured the expression of endogenous pluripotent genes by qRT-PCR. *Gbx2, lifR, Klf2*, and *DNMT3L* were upregulated in iCSCs, while expression of *Nanog* and *Oct4* increased only slightly in shMBD3-iCSCs (Figure 1E). However, immunofluorescence staining showed that NANOG was expressed in iCSCs (Figure 1F). Therefore, C3A-shMBD3, C3A-shCtrl and C3A were reprogrammed into stem-like cancer cells.

### shMBD3-iCSCs acquired pluripotency and properties of CSCs

**Figure 2:**
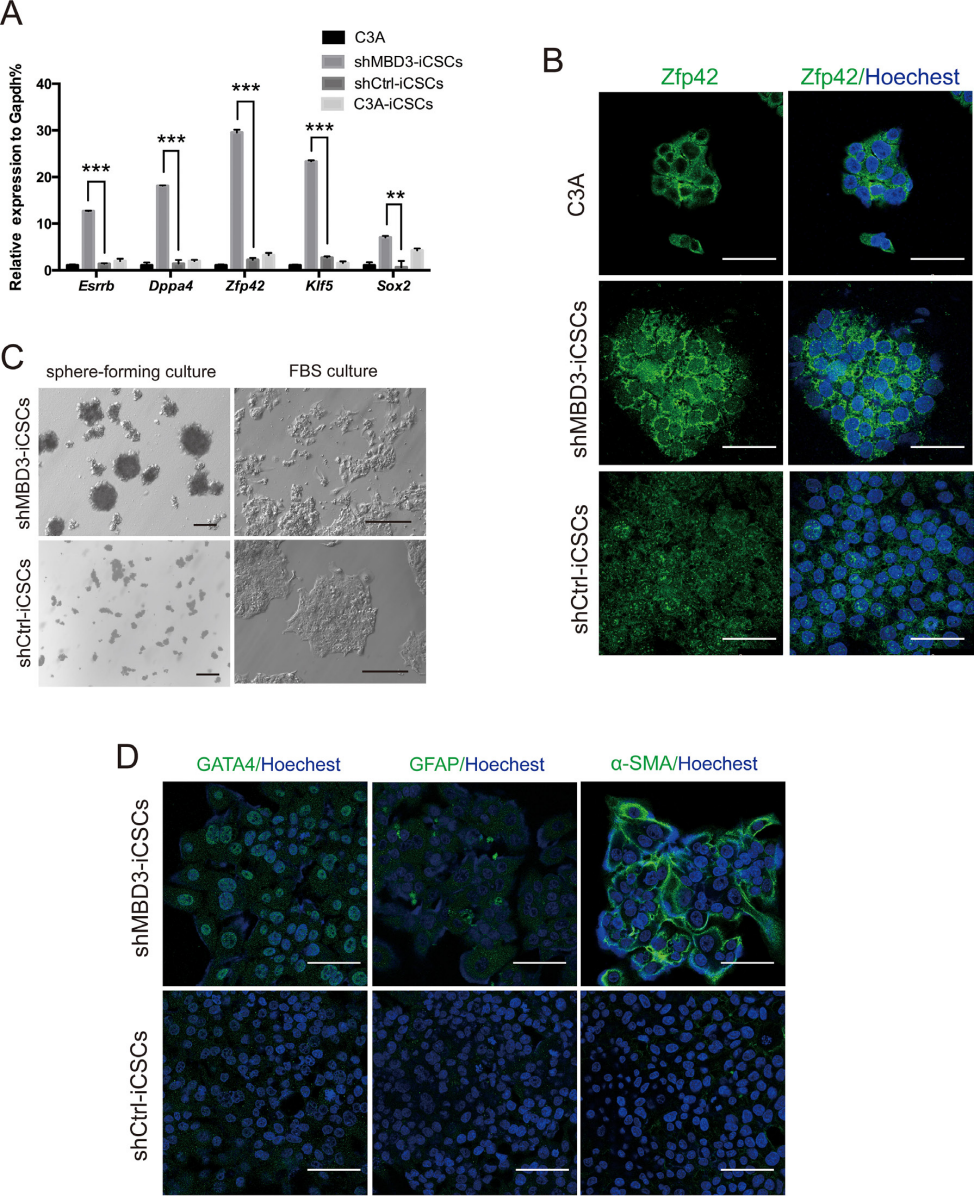
shMBD3-iCSCs acquired pluripotency **A**. Relative expression of *Esrrb, Dppa4, Zfp42, Klf5* and *Sox2* in C3A cells, shMBD3-iCSCs, shCtrl-iCSCs and C3A-iCSCs by RT-qPCR. (n=3; **p<0.01,***p<0.001) **B**. Immunostaining of Zfp42 in C3A cells, shMBD3-iCSCs and shCtrl-iCSCs. Scale bars, 50μm. **C**. Representative phase-contrast images of the different morphology of differentiated shMBD3-iCSCs and shCtrl-iCSCs. Scale bars, 100μm. **D**. Immunostaining of mesoderm marker alpha-SMA, ectoderm marker GFAP and endoderm marker GATA4 in differentiated shMBD3-iCSCs and shCtrl-iCSCs. Scale bars, 75μm.

We found that *Esrrb, Dppa4, Sox2, Zfp42* and *Klf5* were markedly increased in shMBD3-iCSCs, whereas they were almost unchanged in shCtrl-iCSCs and C3A-iCSCs (Figure 2A). Immunofluorescence staining further demonstrated that Zfp42 was highly expressed in nucleus and cytoplasm in shMBD3-iCSCs, while it was expressed only in cytoplasm in C3A cells (Figure 2B). The upregulation of these pluripotent genes indicated that shMBD3-iCSCs obtained pluripotency. Since CSCs could differentiate into various cell types [9], we investigated the iCSCs differentiation capacity. Dissociated iCSCs were suspended in iCSCs medium without bFGF for 21 days and then cultured in FBS containing medium for 7 days. We detected that shMBD3-iCSCs differentiated into a variety of cellular morphologies (Figure 2C). Immunofluorescence staining showed that shMBD3-iCSCs had differentiated and expressed endoderm marker GATA4, ectoderm marker GFAP and mesoderm marker alpha-SMA, but shCtrl-iCSCs were unable to express these differentiation markers (Figure 2D). Thus, shMBD3-iCSCs acquired pluripotency and were able to differentiate.

**Figure 3:**
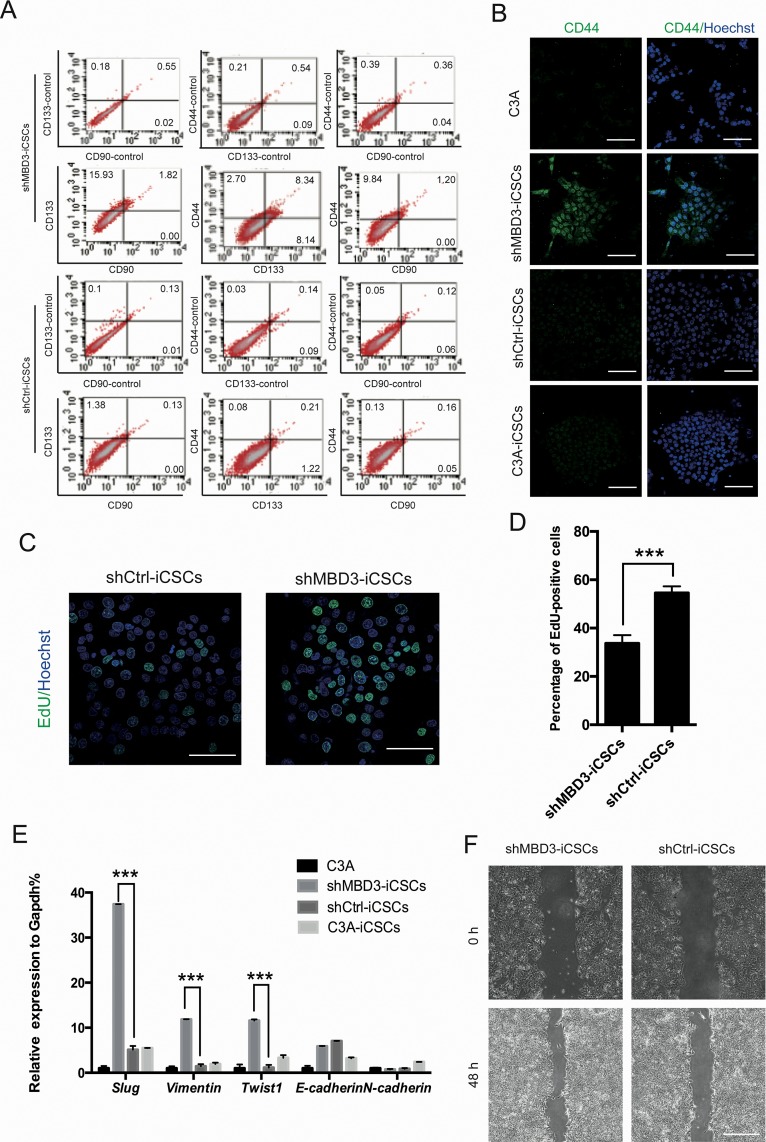
shMBD3-iCSCs have properties of CSCs **A**. Flow cytometric analysis of liver CSCs markers CD44, CD133 and CD90 in shMBD3-iCSCs and shCtrl-iCSCs. Numbers indicate the percentage of positive cells. **B**. Immunostaining of CD44 in C3A cells, shMBD3-iCSCs, shCtrl-iCSCs and C3A-iCSCs. Scale bars, 100μm. **C**. Immunostaining of EdU-positive cells in shMBD3-iCSCs and shCtrl-iCSCs. Scale bars, 75μm. **D**. Percentage of EdU-positive cells in shMBD3-iCSCs and shCtrl-iCSCs. (n=6; ***p<0.001) **E**. Relative expression of EMT-related genes *Slug, Vimentin, Twist1, E-cadherin* and *N-cadherin* in C3A cells, shMBD3-iCSCs, shCtrl-iCSCs and C3A-iCSCs. (n=3; ***p<0.001) **F**. Wound-healing assay performed in shMBD3-iCSCs and shCtrl-iCSCs. Representative images are shown at indicated time points. Scale bars, 200 μm.

Next, we analyzed liver CSCs characteristics in iCSCs. We tested three liver CSCs markers CD133, CD44, and CD90 by flow cytometry analysis, which showed that shMBD3-iCSCs had higher levels of CD133 and CD44, while CD90 was not expressed in iCSCs (Figure 3A). However, since merely a fraction of shMBD3-iCSCs expressed CD133 or CD44, MFI ratio showed only slight differences (Supplementary Figure 1A). Consistently, we found that CD44 was located in nucleus in shMBD3-iCSCs (Figure 3B), indicating the CSCs properties [10]. We also analyzed the proliferation of iCSCs. The EdU cell proliferation assay showed that shMBD3-iCSCs had a lower proliferative state than shCtrl-iCSCs (Figure 3C, 3D), suggesting the stem-like properties of shMBD3-iCSCs. The results from flow cytometry analysis using CFSE staining showed consistent properties (Supplementary Figure 1B). We also analyzed the epithelial-mesenchymal transition (EMT) markers in iCSCs, since previous studies showed that EMT-related transcription factors could induce a CD44-high/CD24-low pattern on epithelial cells, which was associated with somatic cells obtaining stem cell and CSCs properties [11]. The results of qRT-PCR showed that mesenchymal marker *N-cadherin* was almost unchanged in iCSCs, and epithelial marker *E-cadherin* was slightly upregulated. Notably, mesenchymal markers *Twist1, Slug* and *Vimentin* were markedly increased in shMBD3-iCSCs (Figure 3E), indicating that shMBD3-iCSCs obtained the potential of invasion and metastasis. We further investigated the invasion ability of iCSCs by wound-healing assay, which confirmed that shMBD3-iCSCs acquired invasion phenotype (Figure 3F and Supplementary Figure 1C). Therefore, shMBD3-iCSCs acquired more CSCs properties.

### Upregulated c-JUN in shMBD3-iCSCs activates pluripotent genes and induces EMT

**Figure 4:**
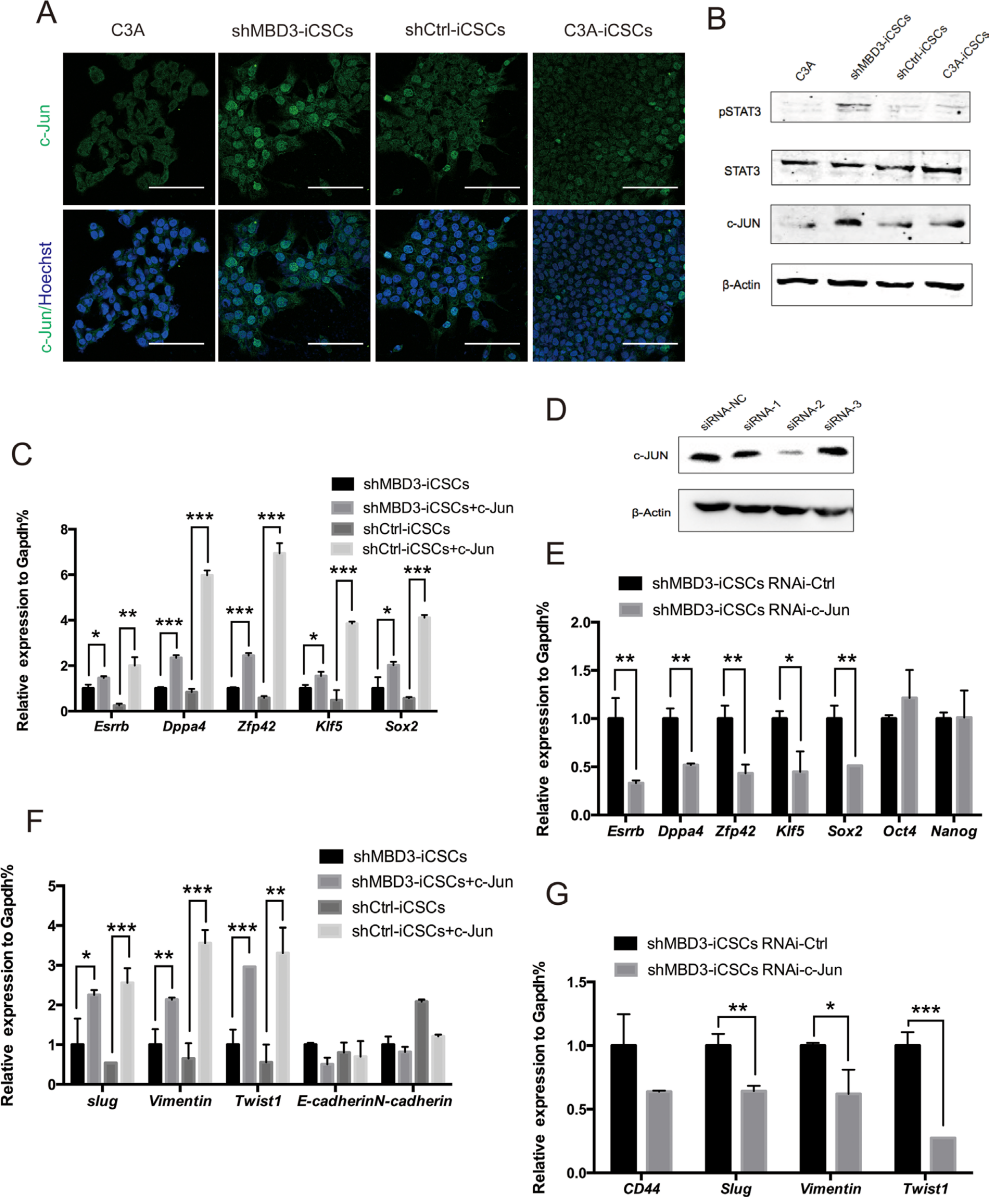
c-JUN induces pluripotent genes and EMT-related genes **A**. Immunostaining of c-JUN in C3A cells, shMBD3-iCSCs, shCtrl-iCSCs and C3A-iCSCs. Scale bars, 100μm. **B**. Immunoblotting of pSTAT3, STAT3 and c-JUN in C3A cells, shMBD3-iCSCs, shCtrl-iCSCs and C3A-iCSCs. **C**. Relative expression of *Esrrb, Dppa4, Zfp42, Klf5* and *Sox2* in shMBD3-iCSCs, shMBD3-iCSCs overexpressed c-JUN, shCtrl-iCSCs and shCtrl-iCSCs overexpressed c-JUN by RT-qPCR. (n=3; *p<0.05, **p<0.01,***p<0.001) **D**. Immunoblotting analysis of c-JUN knockdown in shMBD3-iCSCs by three siRNA. Scramble siRNA was used as negative control (NC). **E**. Relative expression of *Esrrb, Dppa4, Zfp42, Klf5, Sox2, Oct4* and *Nanog* in shMBD3-iCSCs transfected with c-JUN siRNA-2 or siRNA-NC by RT-qPCR. (n=3; *p<0.05, **p<0.01) **F**. Relative expression of EMT-related genes *Slug, Vimentin, Twist1, E-cadherin* and *N-cadherin* in shMBD3-iCSCs, shMBD3-iCSCs overexpressed c-JUN, shCtrl-iCSCs and shCtrl-iCSCs overexpressed c-JUN by RT-qPCR. (n=3; *p<0.05, **p<0.01,***p<0.001) **G**. Relative expression of *CD44* and EMT-related genes *Slug, Vimentin, Twist1* in shMBD3-iCSCs transfected with c-JUN siRNA-2 or siRNA-NC by RT-qPCR. (n=3; *p<0.05, **p<0.01,***p<0.001).

The fact that repression of MBD3 upregulated expression of pluripotent genes and CSCs-related genes suggested that MBD3 might mediate reprogramming by modulating critical downstream genes, which could trigger upregulation of associated genes. Since the transcription factor c-JUN binds to *Nanog* promoter to express higher level of stem-like genes in human colorectal cancer [12], and MBD3 can repress *c-Jun* transcription in colon cancer cells [13], we analyzed c-JUN expression in iCSCs. Protein expression of c-JUN was increased in shMBD3-iCSCs (Figure 4A). Consistently, STAT3 was phosphorylated in shMBD3-iCSCs (Figure 4B), which can be induced by c-JUN/JNK pathway [14].

We then tested the regulatory role of c-JUN in the induction of pluripotent genes in shMBD3-iCSCs. Overexpression of c-JUN in shMBD3-iCSCs and shCtrl-iCSCs induced expression of pluripotent genes *Esrrb, Dppa4, Zfp42, Klf5* and *Sox2* (Figure 4C). Inhibition of c-JUN by RNAi in shMBD3-iCSCs reduced the expression of these genes (Figure 4D, 4E). Consistently, *Twist1, Slug* and *Vimentin* were upregulated by overexpressed c-JUN in shMBD3-iCSCs and shCtrl-iCSCs and downregulated by inhibition of c-JUN in shMBD3-iCSCs (Figure 4F, 4G). However, we were unable to demonstrate a significant effect of c-JUN on CD44 expression, and could not detect c-JUN binding in close proximity to CD44 loci by ChIP-qPCR. These results suggest that c-JUN plays a positive regulatory role in shMBD3-iCSCs, in accordance with the fact that c-JUN is transcriptional activator instead of repressor.

### Without repression of MBD3/NuRD, c-Jun directly activates pluripotent genes

**Figure 5:**
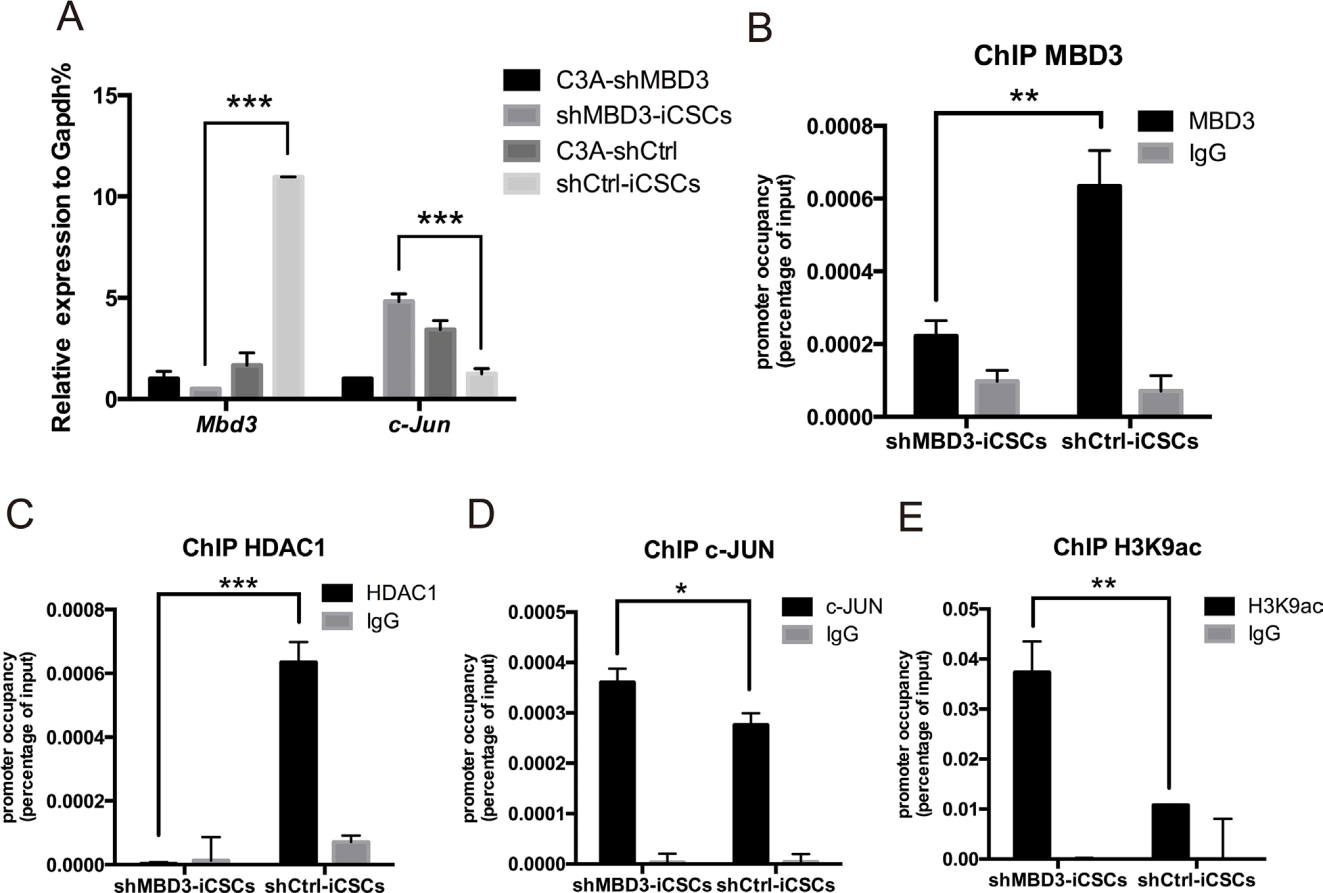
MBD3/NuRD inhibits c-Jun transcription **A**. Relative expression of *Mbd3* and *c-Jun* in C3A-shMBD3 cells, shMBD3-iCSCs, C3A-shCtrl and shCtrl-iCSCs cells. (n=3; ***p<0.001) **B**. ChIP-qPCR for the AP-1 site on the *c-Jun* promoter using MBD3-specific antibodies. (n=3; **p<0.01,***p<0.001) **C**. ChIP-qPCR for the AP-1 site on the *c-Jun* promoter using HDAC1-specific antibodies. (n=3; **p<0.01,***p<0.001) **D**. c-JUN ChIP-qPCR for the AP-1 site on the *c-Jun* promoter using c-JUN-specific antibodies. (n=3; *p<0.05) **E**. ChIP-qPCR for acetylated histone 3 K9 (H3K9ac) for the AP-1 site on the *c-Jun* promoter. (n=3; **p<0.01).

To investigate whether MBD3 inhibits CSCs properties through c-JUN, we first analyzed expression of MBD3 and c-JUN. *Mbd3* expression increased, while *c-Jun* expression decreased after reprogramming in C3A-shCtrl. In contrast, reprogramming of C3A-shMBD3 into shMBD3-iCSCs upregulated *c-Jun* expression (Figure 5A), indicating an inverse correlation between MBD3 and c-JUN in iCSCs. Analysis of MBD3 binding to *c-Jun* promoter by ChIP revealed MBD3 binding to AP-1 site in the *c-Jun* promoter (Figure 5B). In addition, we found that histone deacetylase 1 (HDAC1), another subunit in the NuRD complex, was bound to the same region in shCtrl-iCSCs (Figure 5C), suggesting that the MBD3/NuRD complex represses *c-Jun* transcription [13]. In contrast, ChIP of c-JUN on the AP-1 sites demonstrated that c-JUN was recruited more efficiently to the AP-1 sites in shMBD3-iCSCs (Figure 5D). Since H3K9 acetylation increases transcription, we analyzed H3K9ac recruitment to *c-Jun* promoter. As shown in Figure 5E, the AP-1 sites in the *c-Jun* promoter exhibited higher level of H3K9 acetylation in shMBD3-iCSCs. Thus, without repression of MBD3/NuRD, c-JUN binds to AP-1 promoter sites and activates c-JUN transcription.

**Figure 6:**
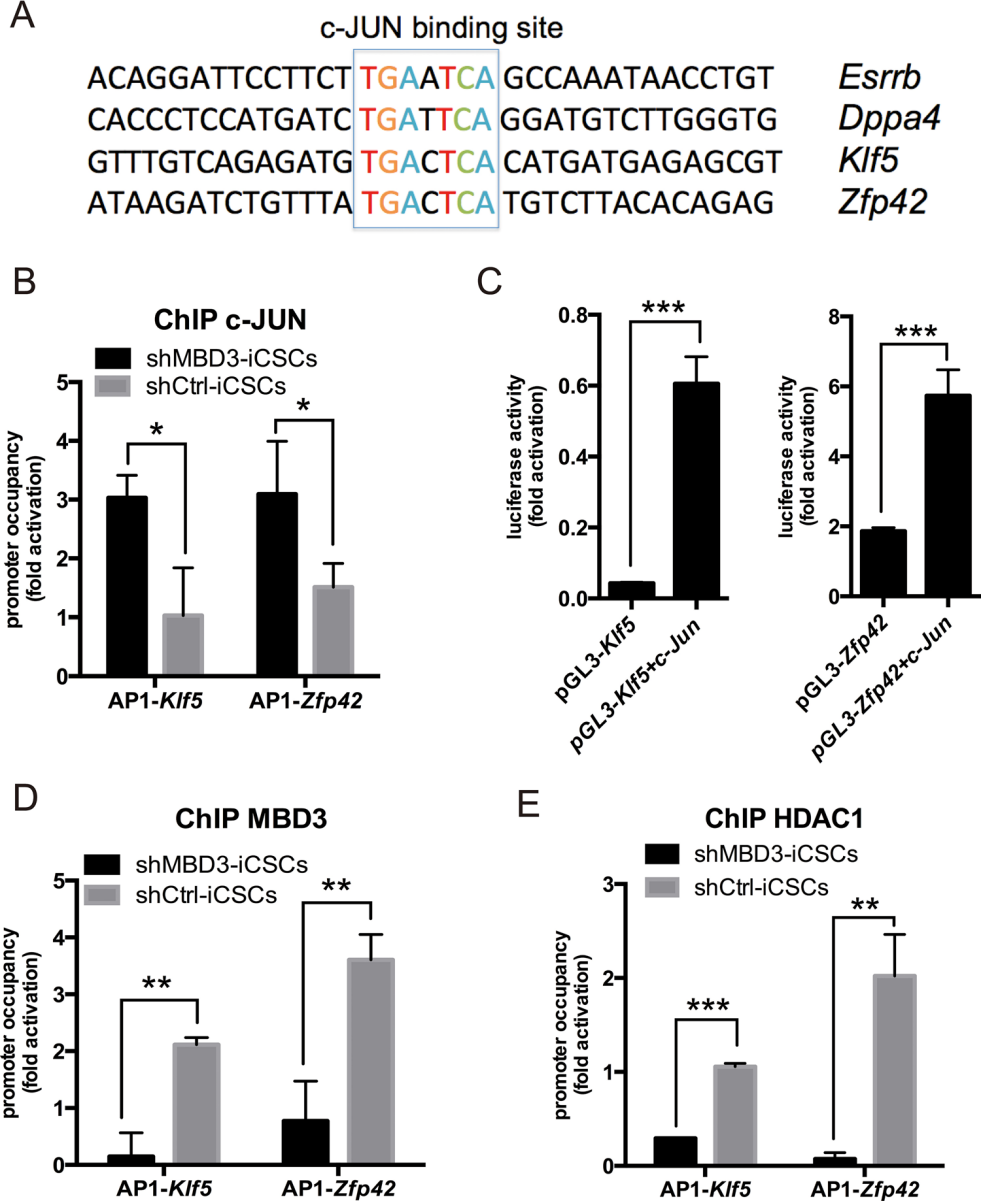
c-JUN induces pluripotent genes directly in shMBD3-iCSCs **A**. Presence of a consensus c-JUN-binding sequence of indicated pluripotent genes. **B**. ChIP-qPCR for the c-JUN-binding sites on the *Klf5* and *Zfp42* promoters using c-JUN-specific antibodies. (n=3; *p<0.05) **C**. c-JUN enhanced *Klf5* and *Zfp42* reports. pGL3-based reports and TK-Renilla were co-transfected with c-Jun or vector plasmids into 293T cells. Luciferase activity was detected at 48 h post-transfection. (n=3; ***p<0.001) **D**. MBD3 ChIP-qPCR for the c-JUN-binding sites on the *Klf5* and *Zfp42* promoters. (n=3; **p<0.01,***p<0.001) **E**. HDAC1 ChIP-qPCR for the c-JUN-binding sites on the *Klf5* and *Zfp42* promoters. (n=3; **p<0.01,***p<0.001).

Next, we investigated whether c-JUN could bind to pluripotent genes directly as a transcriptional activator. We found a canonical c-JUN/AP-1-binding motif TGAXTCA in the promoter regions of many pluripotent genes induced by c-JUN (Figure 6A). As shown in Figure 6B, c-JUN was recruited to AP-1 sites in *Klf5* and *Zfp42* promoters in shMBD3-iCSCs. We used luciferase reporter containing the AP-1 sites to confirm the activation of c-JUN on *Klf5* and *Zfp42* promoters. We found that c-JUN increased 14- and 3-fold *Klf5* and *Zfp42* reporter activities, respectively (Figure 6C). Considering that MBD3/NuRD can bind to AP-1 sites and repress *c-Jun* transcription, we analyzed MBD3 and HDAC1 recruitment to AP-1 sites in *Klf5* and *Zfp42* promoters. As shown in Figure 6D and 6E, MBD3 and HDAC1 indeed bound to *Klf5* and *Zfp42* promoters in shCtrl-iCSCs, suggesting that NuRD represses *Klf5* and *Zfp42* transcription. Together, our results indicate that c-JUN activates transcription of *Klf5* and *Zfp42*, while NuRD represses transcription of these genes.

## DISCUSSION

In this study, we have identified a suppressor role of MBD3/NuRD in generation of stemness and CSCs properties in iCSCs. In addition, we have found that c-JUN is increased in iCSCs when MBD3 is inhibited, resulting in increased expression of CSCs-related genes.

Our results are in accordance with recent studies that indicated the inhibitory role of MBD3 in reprogramming somatic cells into IPSC [6, 7], although other study suggested that MBD3 facilitates generation of IPSC [8]. It seems that the reprogramming conditions could influence the role of MBD3, but the signaling pathways and transcription factors involved remain elusive. Our data indicates inhibitory role of MBD3 in CSCs properties. In addition, our results suggest that NuRD represses c-Jun transcription directly, while c-JUN could activate transcription of pluripotent genes. Although the reprogramming mechanisms of somatic cells and cancer cells are distinct, our results elucidate the function of MBD3 and c-JUN in reprogramming.

We also found that c-JUN facilitates expression of pluripotent genes, such as *Klf5, Zfp42, Dppa4* and *Esrrb*, which is consistent with previous observations that c-JUN plays a pivotal role in the maintenance of self-renewal in cancer stem cells [15, 16]. Moreover, Klf5 is essential for oncogenesis and can strengthen drug resistance in CSCs [17–19]. Zfp42 belongs to Myc-centered regulatory network, which is active in various cancers [20]. In our experiments, depletion of MBD3 enabled c-JUN to activate *Klf5* and *Zfp42* transcription in shMBD3-iCSCs, whereas MBD3/NuRD could bind to the AP-1 sites in *Klf5* and *Zfp42* promoters.

Our results are in apparent disagreement with the recent study by Liu *et al*., that suggested an inhibitory role for c-JUN in reprogramming [21]. There are several differences between our study and the study by Liu *et al*, including the choice of cells and reprogramming conditions. In contrast to our study, Liu *et al*. used mouse embryonic fibroblasts (MEFs) to generate iPSCs. In liver cancer, c-JUN forms a positive feedback loop with pluripotent genes to expedite cancer stemness [22]. However, the signaling network of c-Jun may be significantly different in MEFs [23]. Moreover, NuRD can interact with pluripotent genes and c-Jun in various ways [13, 24–26], and thus depletion of MBD3 can influence the interaction between c-Jun and pluripotent genes. As a member of the AP-1 family, c-JUN can dimerize with other AP-1 members to generate diverse transcription complexes. Thus, more information about the involvement of other AP-1 members, such as JunB, c-Fos, Fra1 and Fra2, in shMBD3-iCSCs is needed.

Previous studies have shown that NuRD represses transcription by binding to methylated DNA, mediating heterochromatin formation, and transcriptional silencing [27, 28]. NuRD can also repress expression of pluripotent genes by opposing transcriptional activators. A previous study has shown that NuRD is recruited to *Nanog* by the transcriptional repressor Zfp281, and inhibits expression of pluripotency transcription factors, such as Klf4, Klf5, Zfp42, and Tbx3 in ESCs [24]. Therefore, NuRD might repress *Klf5, Zfp42* and other pluripotent genes directly or indirectly; more data are needed to dissect the mechanism. In the future, we will investigate the network between MBD3 and c-JUN and their regulatory mechanisms in iCSCs.

CD133 and CD44 are CSCs markers, which are involved in invasion, metastasis and chemotherapy resistance [29–32]. In our study, we have found that CD44 is highly expressed in the nucleus in shMBD3-iCSCs. It has been reported that nuclear translocation of CD44 can reprogram colon cancer cells into CSCs [10]. In addition, CD44 is required for stemness properties and might induce EMT [33]. In somatic cells reprogramming, c-JUN upregulates CD44 and induces EMT [21]. We showed that the induced EMT-related genes in shMBD3-iCSCs could be activated by c-JUN, but we were unable to detect any influence of c-JUN on CD44. Our results suggest that c-JUN activates CSCs-related genes in liver cancer stem cells, but more studies are needed to understand the network of MBD3, c-JUN, EMT and CD44.

In summary, we have demonstrated that MBD3/NuRD inhibits formation of liver iCSCs. In addition, we have shown that MBD3 suppression induces c-JUN, resulting in the induction of pluripotent genes in iCSCs. Therefore, our study provides a novel way to establish liver CSCs to study the mechanisms of liver cancer development. Furthermore, our study indicates that c-JUN serves as a potential therapeutic target for liver cancer.

## MATERIALS AND METHODS

### Plasmids

Tet-O-FUW-Pou5f1, -Sox2, -Klf4, and -c-Myc retroviral vectors (Addgene, Cambridge, USA) were designed with a tetracycline responsive element. The plasmids were co-transfected with pCMV-Gag-Pol and pCMV-VSVG. Lentivirus small hairpin RNA (shRNA) vectors are based on pll3.7 (Addgene, Cambridge, MA), in which the green fluorescent protein (GFP) coding region was removed. The plasmids encoding shMBD3-1 (sense, 5'-gatgctgatgagcaagatg-3') and shMBD3-2 (sense, 5'-gccttcatggtgaccgacg-3') were co-transfected with pMD2.G and pSPAX2. pGL3-basic plasmids containing the *Klf5* and *Zfp42* promoter region, pmcherry-N1-c-Jun, pEGFP-N1-MBD3 and pHBLV-c-Jun were synthesized by Polepolar (Beijing, China).

### Cell culture

All cells were cultured at 37°C with 5% CO_2_. C3A cells were derived from the human hepatoma cell line HepG2. The culture medium for C3A cells was Eagle's minimum essential medium (Gibco, New York) containing 10% fetal bovine serum (HyClone, Logan), 0.1 mM non-essential amino acids (Gibco, New York), and 0.1 mM 2-mercaptoethanol (Gibco, New York). Culture medium for iCSCs cells was Dulbecco's modified Eagle's medium (DMEM)/Ham's F-12 medium (Gibco, New York) containing 20% knockout serum replacement (Gibco, New York), 1 mM L-glutamine, 0.1 mM nonessential amino acids, 0.1 mM 2-mercaptoethanol, and 10 ng/ml recombinant human basic fibroblast growth factor (bFGF; Life Technologies, New York). Suspension culture medium was iCSCs medium without bFGF. All iCSCs cells were maintained on Matrigel (BD Biosciences, Franklin Lakes) and passaged with 0.5 mM EDTA.

### Lentiviral infection

The day before transfection, 293T cells were seeded at 8×10^6^ cells per 100 mm dish. On the next day, retroviral vectors were introduced into 293T cells using VigoFect transfection reagent (Vigorous Biotechnology, China) according to the manufacturer's recommendations. 24 hours after transduction, the medium was replaced. After 24 h, virus-containing supernatants derived from these 293T cultures were filtered through a 0.45 mm cellulose acetate filter. The viruses were then concentrated by ultracentrifugation and transduced into C3A cells in medium containing 10 ng/ml polybrene. Target cells were incubated in the virus/polybrene-containing supernatants for 4 h to overnight. After infection, the cells were replaced in 10 ml fresh medium. For stably transfected C3A, three days after infection, we added G418 at a final concentration of 0.6 to 0.8 mg/ml for 2 weeks. G418-resistant cells were further cultured in the presence of 0.4 mg/mL G418.

### Generation of iCSCs

The day before infection, C3A-shMBD3, C3A-shCtrl and C3A cells were seeded at 6×10^4^ cells per well in 24 wells cell culture cluster. For iCSCs induction, C3A-shMBD3, C3A-shCtrl and C3A cells were infected with retroviruses expressing Oct4, Sox2, Klf4, c-Myc and cultured in iCSCs culture medium supplemented with 20 ng/ml doxycycline (SIGMA-ALDRICH, St. Louis) for 20 days on vitronectin-coated dishes. On day 21, single colonies were picked and passaged in iCSCs culture medium on Matrigel-coated dishes.

### *In vitro* differentiation of iCSCs

Adherent iCSCs colonies were dissociated into clumps with Accutase (Life Technologies, New York). The produced clumps were transferred to low attachment culture dishes in iCSCs medium without bFGF. After suspension culture for 21 days, iCSCs were passaged onto gelatin-coated dishes and incubated in DMEM containing 10% fetal bovine for another 7 days.

### Immunofluorescence

Cells were fixed with 4% paraformaldehyde. Permeabilization was performed with 0.2% Triton X-100. Primary antibodies included antibodies against NANOG (Abcam), c-JUN (31419, Abcam), REX1 (50828, Abcam), GATA4 (134057, Abcam), and alpha smooth muscle actin (124964, Abcam). Secondary antibodies were Alexa Fluor^®^488/594 goat anti-rabbit/mouse IgGs (Origene, Rockville). Counter staining was performed with Hoechst 33342. Images were captured under a TCSSP5 Confocal Microscope (Leica, Buffalo Grove, USA). Merged images were obtained according to the recommended procedure using the Leica software.

### Western blot analysis

Primary antibodies used were: c-JUN (31419, Abcam), MBD3 (157464, Abcam) and beta-actin (PM053, MBL). Images were captured by the Odyssey Fluorescent Western Scanning System (LI-COR, Lincoln, USA). Data were analyzed with the Odyssey Imaging System.

### RNA isolation, RT-PCR, and semi-quantitative real-time PCR

Total RNA was extracted using Trizol (Life Technologies, New York). cDNA synthesis was performed with a M-MLV Reverse Transcriptase kit (Promega, Madison) in accordance with the manufacturer's instructions. RT-PCR was performed with ExTaq (Takara Bio). Real-time PCR was performed with GoTaq^®^ qPCR Master Mix (Promega, Madison). Primer sequences are listed in Supplementary Table 1. Signals were detected with Mx3000P and Mx3005P QPCR Systems (Agilent Technologies, Santa Clara, USA).

### Flow cytometry

Cells were harvested with accutase (Life Technologies, New York), and then suspended (2 × 10^6^ cells/100 μl) in staining buffer. PE-Cy7^®^-conjugated CD44 (Life Technologies, New York), CD133/1(AC133)- PE (Miltenyi Biotec, Germany), and fluoresceinisothiocyanate-anti human CD90 (BD Biosciences, Franklin Lakes) antibodies were added to the cell suspension at the concentrations recommended by the manufacturer and incubated at 4°C in the dark for 60 min. PE-Cy7^®^-IgG2b (Invitrogen), PE-IgG1 (Miltenyi Biotec, Germany) and FITC-IgG2a (BD Biosciences, Franklin Lakes) were used as isotype controls. Carboxyfluorescein succinimidyl ester (Dojindo, Japan) was used for CFSE staining. Signals were detected with a FACSCalibur flow cytometer (BD Biosciences, Franklin Lakes).

### Luciferase assay

Transfections were carried out in 293T cells using VigoFect transfection reagent (Vigorous Biotechnology, China). Cells that were transfected with the expression vectors pHBLV-c-JUN and the reporter vectors were harvested 48 h after transfection. Reporter activities were measured by using the dual-luciferase reporter assay system (Promega). Each assay was performed in duplicate, and all results are shown as mean ± SD for at least three independent assays.

### RNA interference (RNAi)

c-JUN siRNA oligonucleotides c-JUN(1)(sense, 5′-GCAAAGAUGGAAACGACCU-3′), c-JUN(2) (sense, 5′-CCUUCUAUGACGAUGCCCU-3′), and c-JUN(3) (sense, 5′-GAUGGAAACGACCUUCUAU-3′) were synthesized by GenePharma (Shanghai, China). The cells were harvested 48 h after transfection. Western blot assay was used to evaluate the RNAi effect.

### ChIP-qPCR

ChIP was performed following the cross-linking chromatin immunoprecipitation protocol (http://www.abcam.cn/protocols/cross-linking-chromatin-immunoprecipitationx-chip-protocol). Antibodies used included c-JUN (31419, Abcam), MBD3 (157464, Abcam), HDAC1 (31263, Abcam) and H3 (acetyl K9) (10812, Abcam). Real-time PCR primers are listed in Supplementary Table 1.

### EdU cell proliferation assay

EdU cell proliferation assay was performed using EdU HTS Kit 488 (SIGMA-ALDRICH, St. Louis). Cells were exposed to EdU for 30 min.

### Statistical analysis

All data are expressed as the mean ± SD from at least three independent experiments. The Student's two-tailed t-test with significance set at p<0.05 was used to determine statistical significance for each assay. Error bars indicate standard deviation.

## SUPPLEMENTARY MATERIALS FIGURES AND TABLES


