# Supplementary material for: MBD3 inhibits formation of liver cancer stem cells
Source: Oncotarget. 2016 Nov 22;8(4):6067–78. doi: 10.18632/oncotarget.13496 (PMC5351613; doi:10.18632/oncotarget.13496)
Supplement: Supplementary file 1 [file oncotarget-08-6067-s001.pdf]

MBD3 inhibits formation of liver cancer stem cells

SUPPLEMENTARY FIGURE AND TABLE

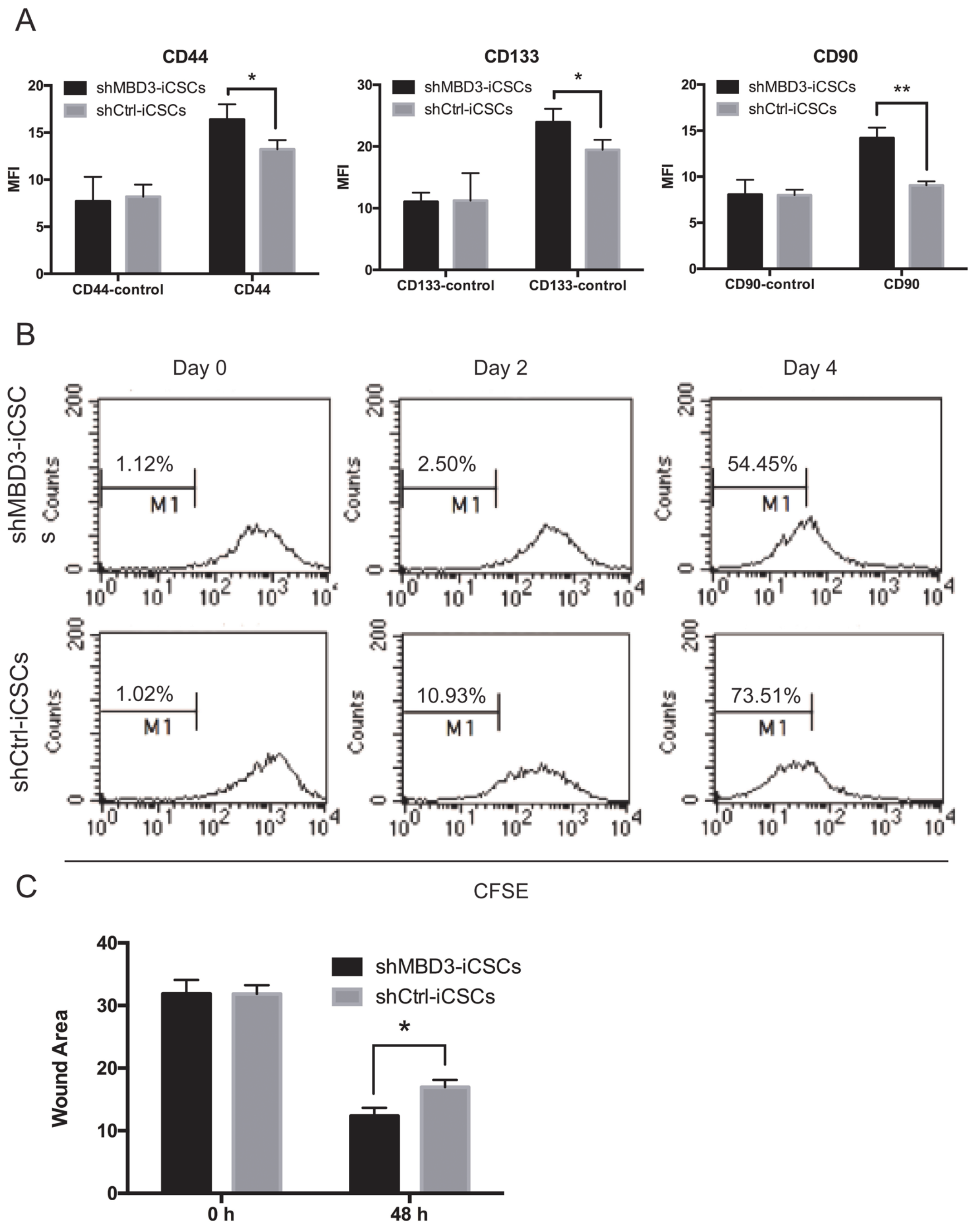

Supplementary Figure 1: A. Median of fluorescence intensity of CD44, CD133 and CD90 in shMBD3-iCSCs and shCtrl-iCSCs. (n=3; *p<0.05, **p<0.01) **B.** Flow cytometric analysis of CFSE staining in shMBD3-iCSCs and shCtrl-iCSCs. **C.** Quantities analysis of wound-healing assay performed in shMBD3-iCSCs and shCtrl-iCSCs. (n=3; *p<0.05).

Supplementary Table 1: Primers used for qRT-PCR and ChIP-qPCR

	Forward primer (5`-3`)	Reverse primer (5`-3`)
**exo *Oct4***	ATGGGGAAAGAAGCTCAGTG	GGCATTAAAGCAGCGTATCC
**exo *Sox2***	GCCCCTGTCGCACATGT	GGCATTAAAGCAGCGTATCC
**exo *Klf4***	TGCCTTACACATGAAGAGGCAC	GGCATTAAAGCAGCGTATCC
**exo *c-Myc***	ACAGCTTCGAAACTCTGGTGC	GGCATTAAAGCAGCGTATCC
**endo *Oct4***	GGGAGATTGATAACTGGTGTGTT	GTGTATATCCCAGGGTGATCCTC
**endo *Sox2***	GGGAAATGGGAGGGGTGCAAAAGAGG	TTGCGTGAGTGTGGATGGGATTGGTG
Nanog	CTAAGAGGTGGCAGAAAAACA	CTGGTGGTAGGAAGAGTAAAGG
Gbx2	CCGCCTTCAGCATAGACTCG	GGTAGCCGGTGTAGACGAAAT
LifR	TGGAACGACAGGGGTTCAGT	GAGTTGTGTTGTGGGTCACTAA
DNMT3L	TACGACCGAGAGTCGGAGAAT	GCCCAAACTCGTCAGCTCTTT
Klf2	CTACACCAAGAGTTCGCATCTG	CCGTGTGCTTTCGGTAGTG
Zfp42	GCCTTATGTGATGGCTATGTGT	ACCCCTTATGACGCATTCTATGT
Klf5	CCTGGTCCAGACAAGATGTGA	GAACTGGTCTACGACTGAGG
Esrrb	TCGCTGCCCTATGACGACA	CTTCTTGTACCTGCGTACCAG
Dppa4	GACCTCCACAGAGAAGTCGAG	TGCCTTTTTCTTAGGGCAGAG
MBD3	GGCCACAGGGATGTCTTTTACTATAG	GTTGTGGCTTGCTGCGG
c-Jun	TCGACATGGAGTCCCAGGA	GGCGATTCTCTCCAGCTTCC
E-cadherin	TGCCCAGAAAATGAAAAAGG	GTGTATGTGGCAATGCGTTC
N-cadherin	ACAGTGGCCACCTACAAAGG	CCGAGATGGGGTTGATAATG
Vimentin	GAGAACTTTGCCGTTGAAGC	GCTTCCTGTAGGTGGCAATC
Twist1	GGAGTCCGCAGTCTTACGAG	TCTGGAGGACCTGGTAGAGG
Slug	GACTACCGCTGCTCCATT	GAGGAGGTGTCAGATGGA
CD44	CTGCCGCTTTGCAGGTGTA	CATTGTGGGCAAGGTGCTATT
GapdH	TGCCAAATATGATGACATCAAGAA	GGAGTGGGTGTCGCTGTTG
**AP1-*c-Jun***	GCGACGCGAGCCAATG	AGCCCGAGCTCAACACTTATCT
**c-Jun-*Klf5***	AGGGCAGTGGCGTTTGTCAG	AAATGCGTTTCATCTCGGAC
**c-Jun-*Zfp42***	ACCCAGATAAGATCTGTTTA	ATTAGAGCCACTGTCAAAAA
